# A prognostic nomogram for papillary thyroid cancer lymph node metastasis based on immune score

**DOI:** 10.3389/fendo.2022.993856

**Published:** 2022-12-01

**Authors:** Yihua Lu, Kai Qian, Mengjia Fei, Kai Guo, Yuan Shi, Zhuoying Wang

**Affiliations:** Department of Head and Neck Surgery, Shanghai Jiao Tong University School of Medicine Affiliated Renji Hospital, Shanghai, China

**Keywords:** immune score, immune microenvironment, nomogram, lymph node metastasis, papillary thyroid cancer (PTC)

## Abstract

**Background:**

Papillary thyroid cancer (PTC) is the most common subtype of thyroid cancer and is characterized by an overall good prognosis and early-stage lymph node metastasis. The immune microenvironment is believed to play a crucial role in PTC initiation, progression and metastasis. However, to our knowledge, prognostic tools for thyroid cancer metastasis based on immune scores have not been adequately explored. This study aimed to construct a clinical nomogram to predict lymph node metastasis in patients with PTC.

**Methods:**

The genomic data and clinical-pathological characteristics of 447 PTC subjects were obtained from TCGA (The Cancer Genome Atlas data). Logistic regression models were performed for univariate and multivariate analyses to identify significant prediction factors. A prognostic nomogram was built based on the multivariate analysis results. The concordance index (C-index) and calibration curve were used to assess the predictive accuracy and discriminative ability of the model.

**Results:**

The patients were divided into two subgroups based on immune scores. We found that patients with high immune scores had significantly higher lymph node metastasis risks (OR and 95% confidence interval [CI]: 1.774[1.130-2.784]) than those with low immune scores. The C-index for lymph node metastasis was 0.722 (95% CI, 0.671‐0.774), which had a favorable performance for clinical prediction. The calibration curve for lymph node metastasis showed significant agreement between the nomogram prediction and actual observation.

**Conclusion:**

High immune scores are significantly correlated with higher lymph node metastasis risk in patients with PTC. Immune score-based prognostic nomograms may help to predict lymph node metastasis and have potential clinical application possibilities.

## Introduction

Thyroid cancer is the most common malignancy in the endocrine system, and the incidence has steadily increased in most countries over the last three decades, with 586,000 new cases in 2020 (http://gco.iarc.fr). Thyroid cancer is now the fastest-growing malignancy worldwide (1, 2) and is mainly associated with the gradual rise in the number of papillary thyroid cancers (3). However, mortality rates have generally shown a stable or decreasing trend and vary significantly between countries. It was reported that the incidence and mortality of thyroid cancer were 9.61/10^5^ and 0.35/10^5^, respectively, in China in 2015, and women have been found to have a higher incidence and mortality than men of all ages (4, 5).

Papillary thyroid cancer (PTC) is the most common histological type of thyroid cancer (6) and the only subtype with a growing incidence in all countries. In many Asian countries, the incidence of PTC has continued to increase more than threefold in the past decade, likely due to the development and application of detection techniques such as ultrasound, CT and MRI and changes in health concepts (1, 5). The vast majority of PTC patients have slow progression and an overall favorable prognosis with a 10-year survival rate of >90% (5). Meanwhile, studies have reported that PTC has a higher risk of early lymph node metastasis, as 20%-90% of PTC patients have lymph node metastasis at the time of initial diagnosis, and the risk of lymph node metastasis and recurrence is as high as 50% and 20%, respectively (7).

Tumor cells and their environment together constitute the tumor microenvironment (TME), which consists of tumor cells, immune cells, stromal cells, microvasculature, and various chemokines and cytokines. In recent years, a growing body of research has shown the association between the TME and tumor cells, especially the crucial role of immune cells in tumor initiation, progression and metastasis (8, 9). Some studies have revealed that immune infiltration is associated with the malignancy level, recurrence and overall survival of PTC (8, 10, 11). Several studies have clarified that immunotherapy, represented by immune checkpoint inhibitors, has a considerable commitment in the treatment of advanced thyroid cancer (12, 13). Challenges remain with respect to identifying PTC patients who could benefit most from immunotherapy. Additionally, the overall infiltration of immune cells in tumor tissue could be estimated from gene expression data by the ESTIMATE algorithm (14), which produces the immune score. Specific immune cells, such as dendritic cells, mast cells, Tregs (12) and neutrophils (15), are correlated with lymph node metastasis and poor prognosis in thyroid cancer. Yet no study has demonstrated the involvement of immune scores in the prediction of lymph node metastasis in PTC.

This study aimed to evaluate the correlation between the immune score and clinical-pathological features of PTC, especially lymph node metastasis, and to construct a nomogram based on the immune score for predicting PTC lymph node metastasis.

## Materials and methods

### Data extraction and processing

The data used in this study were obtained from The Cancer Genome Atlas (TCGA) dataset (16). The TCGA database is the largest open source data platform for oncogenetic analysis, containing genetic and clinical information for over 200 kinds of tumors, as well as the measurement of DNA methylation and RNA sequencing (https://portal.gdc.cancer.gov/). Gene information is mainly TCGA mRNA transcriptome data downloaded *via* USCS Xena (https://xena.ucsc.edu/), with expression data for 19,303 genes from 497 samples. The key variable immune score, representing the immune cell infiltration level, was evaluated.

Corresponding clinical-pathological data were also obtained from USCS Xena (https://xena.ucsc.edu/), which included patient demographic data (e.g., TCGA patient number, age, sex, etc.), tumor-related data (e.g., tumor size, histological type, presence of multifocality, extrathyroid extension, etc.), lymph node metastases, and distant metastases (17, 18).

Inclusion criteria were cases with a pathological diagnosis of papillary thyroid cancer from 1993 to 2013. Exclusion criteria were unknown data on lymphadenopathy status and tumor size. A total of 447 cases could be available for further analysis after the exclusion of duplication.

### Immune score

To evaluate the infiltrating levels of immune cells involved in the tumor microenvironment (TME), the ESTIMATE algorithm (Estimation of STromal and Immune cells in MAlignant Tumors using Expression data) was applied based on the transcriptome data of PTC samples (14). Immune scores were calculated by performing the single-sample gene set enrichment analysis (ssGSEA) from the top-ranked 141 immune signature genes with a specific formula, indicating the sum total score of tumor-infiltrating immune cell enrichment like macrophages, dendritic cells, neutrophils, NK cells, B cells, T cells, mast cells.

### Nomogram

Lymph node metastasis nomograms were formulated based on the results of multivariate regression analysis in R (version 4.1.2; R Foundation for Statistical Computing, Vienna, Austria). The nomogram was subjected to 1000 bootstrap resamples for internal validation of the analyzed database. The performance of the model was evaluated by calculating the concordance index (C-index). The C-index ranges from 0.5 to 1.0, with closer to 1.0 indicating better predictive ability and 0.5 indicating a random chance (19). The discriminatory ability of the model is shown by plotting a calibration curve (20).

### Statistical analysis

The outcome event was lymph node metastasis in any location and number. The cutoff point of the immune score was calculated by SPSS (version 20.0; Illinois, Chicago, USA), whereby the immune score was transformed from a continuous variable to a binary variable.

The following statistics were made use of STATA (version 12.0; Stata Corp, College Station, TX). Correlation analysis of immune score and clinical pathological characteristics was analyzed using Pearson’s *chi*-square test or Fisher’s exact test. Univariate and multivariate logistic regression models were used to identify independent predictors of lymph node metastasis. After the adjustment of clinical-pathological characteristics, odds ratios (ORs) and 95% confidence intervals (CIs) were estimated. All statistical tests were two-sided, and P values of <0.05 were considered statistically significant.

## Results

### Patients’ clinical-pathological characteristics

A total of 447 patients who were pathologically diagnosed with PTC between 1993 and 2013 were included in this study, with a median follow-up time of 943 (535, 1474) days. They were divided into 2 groups according to the presence of lymph node metastases, of which 227 had no lymph node metastases and 220 had lymph node metastases. Among these patients, 122 (27.3%) were male, and 325 (72.7%) were female. The median age of the patients was 46 (36, 58). Pathological T distribution was T1 (29.3%), T2 (31.1%), T3 (34.9%), T4 (4.7%); pathological N distribution was N0 (50.7%), N1 (49.3%); pathological M distribution was M0 (97.8%), M1 (2.2%); overall stage distribution was I (55.7%), II (9.7%), III (23.4%) and IV (11.2%). The median immune infiltration score was 304.05 (-177.092, 913.311), with an optimal cutoff point of 459.93, according to which 191 (42.7%) were divided into a high immune score subgroup and 256 (57.2%) were divided into a low immune score subgroup.

The clinical-pathological characteristics of the different immune infiltration scores are detailed in **Table 1**. The high immune score group tended to have higher overall staging and was more likely to be in stages III-IV than the lower immune score group and a higher proportion of classic PTC.

**Table 1:** Associations between clinical-pathological characteristics and immune scores.

Characteristics	Total	Immune score		χ^2^	P
		<460	≥460		
Total	447	256	191		
Age	447			1.738	0.187
<55	299	163	133		
≥55	158	93	58		
Sex	447			0.209	0.648
Male	122	72	50		
Female	325	184	141		
Pathological T	447			1.062	0.786
T1	131	75	56		
T2	139	84	55		
T3	156	85	71		
T4	21	12	9		
Multifocality	438			0.037	0.848
No	233	132	101		
Yes	205	118	87		
ETE	431			0.713	0.398
No	286	168	118		
Yes	145	79	66		
Pathological M	278			1.984	0.159
M0	272	148	124		
M1	6	5	1		
Radiation exposure	390			0.071	0.79
No	375	212	163		
Yes	15	9	6		
Stage	445			14.416	0.002
I	248	137	111		
II	43	36	7		
III	104	53	51		
IV	50	29	21		
Location	442			0.004	0.947
Unilateral	358	206	152		
Bilateral	84	48	36		
Histological type	376			3.5	0.061
Classical	277	149	128		
Nonclassical	99	64	35		
Loco-regional recurrence	447			0.0174	0.895
No	430	246	184		
Yes	17	10	7		

*ETE, extrathyroid extension.

### Univariate and multivariate regression analysis of lymph node metastasis

Univariate analysis showed that clinical-pathological factors such as immune score, pathological T stage, sex, age, presence of multifocality, extrathyroid extension and histological type were significantly associated with lymph node metastasis (P<0.1), while tumor location and pathological M stage were not predictors of lymph node metastasis in patients with PTC (P>0.1). The results of the univariate analysis are detailed in **Table 2**.

**Table 2:** Univariate analyses of LNM among PTC patients according to clinical-pathological characteristics and immune scores.

	Total	LNM		OR(95% CI)	P value
		LNM(-)	LNM(+)		
Total	447	227	220		
Immune Score					
<460	256	147	109	1	
≥460	191	80	111	0.627(0.247, 1.006)	0.001
Age					
<55	296	138	158	1	
≥55	151	89	62	-0.497(-0.893, -0.100)	0.014
Sex					
Male	122	53	69	1	
Female	325	174	151	-0.406(-0.825, 0.014)	0.058
Pathological T					
T1	131	88	43	1	
T2	139	75	64	0.558(0.063, 1.052)	0.027
T3	156	60	96	1.186(0.699, 1.673)	<0.001
T4	21	4	17	2.163(1.014, 3.312)	<0.001
Multifocality					
No	233	129	104	1	
Yes	205	94	111	0.382(0.005, 0.759)	0.047
ETE					
No	286	167	119	1	
Yes	145	48	97	1.042(0.624, 1.461)	<0.001
Pathological M					
M0	272	145	127	1	
M1	6	3	3	0.133(-1.485, 1.750)	0.872
Radiation exposure					
No	375	187	188	1	
Yes	15	9	6	-0.411(-1.463, 0.642)	0.444
Location					
Unilateral	358	191	167	1	
Bilateral	84	35	49	0.471(-0.010, 0.952)	0.055
Histological type					
Classical	277	133	144	1	
Nonclassical	99	66	33	-0.773(-1.252, -0.293)	0.002

Multivariate logistic regression analysis was performed on six variables, including immune score (low or high), age (<55, ≥55), sex (male or female), pathological T stage (I–IV), presence of multifocality (no or yes) and histological type (classical PTC or nonclassical PTC). The results revealed a significantly increased risk of lymph node metastasis in the high immune score group (OR (95% CI): 1.774 (1.130, 2.784)). In addition, age <55 years old, pathological T stage, unifocal lesion, and nonclassical PTC were associated with a higher risk of lymph node metastasis, whereas gender differences were not significantly associated with lymph node metastasis.

Patients aged ≥55 years had a lower risk of lymph node metastasis than patients with PTC aged <55 years (OR (95% CI): 0.465 (0.280, 0.773)). Patients with stage T2-4 had a significantly increased risk of lymph node metastasis compared to patients with stage T1 (OR (95% CI): 1.898 (1.064, 3.387), 3.705 (2.063, 6.654) and 14.600 (4.123, 51.668) for T2, T3 and T4, respectively). Patients with multifocal lesions (OR (95% CI): 1.595 (1.006, 2.529) and nonclassical histological type (OR (95% CI): 0.370 (0.215, 0.636)) had a higher risk of lymph node metastasis. The results of the multivariate analysis are detailed in **Table 3**.

**Table 3:** Multivariate analyses of LNM among PTC patients according to clinical-pathological characteristics and immune scores.

	OR(95% CI)	P value
Immune score		
<460	1	
≥460	1.774(1.130, 2.784)	0.013
Age		
<55	1	
≥55	0.465(0.280, 0.773)	0.003
Sex		
Male	1	
Female	0.843(0.502, 1.417)	0.52
Pathological T		
T1	1	
T2	1.898(1.064, 3.387)	0.03
T3	3.705(2.063, 6.654)	<0.001
T4	14.600(4.123, 51.668)	<0.001
Multifocality		
No	1	
Yes	1.595(1.006, 2.529)	0.047
Histological type		
Classical	1	
Nonclassical	0.370(0.215, 0.636)	<0.001

### Nomogram

**Figure 1:**
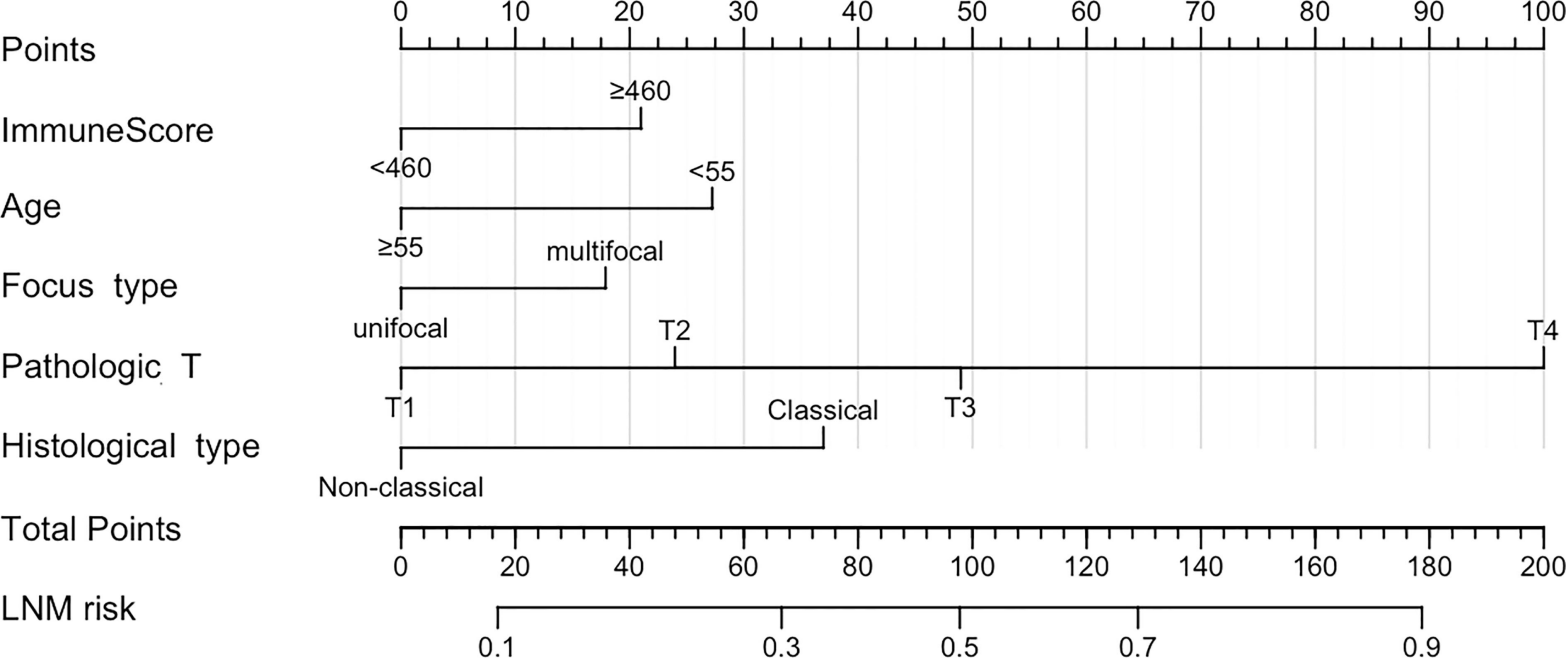
Papillary thyroid cancer nomogram for predicting lymph node metastasis.

**Figure 2:**
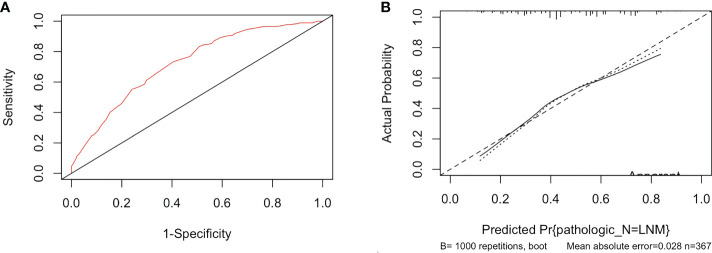
**(A)** The ROC curve of the immune-score-based nomogram. **(B)** The calibration curve of lymph node metastasis for papillary thyroid cancer.

Based on five independent risk factors screened through multivariate logistic regression, we constructed a prognostic model for the risk of lymph node metastasis in patients with PTC (**Figure 1**). The predictive ability of the model was evaluated by the concordance index (0.722 (95% CI: 0.671-0.774)) (**Figure 2A**). The calibration curve for the risk of lymph node metastasis showed good agreement between the predicted and actual observations in the nomogram (**Figure 2B**).

## Discussion

In the current study, we evaluated the predictive significance of the immune score for lymph node metastasis in papillary thyroid cancer. After adjustment for clinical-pathological prognostic factors such as age, sex, T stage, presence of multifocality and histological type, immune scores were significantly associated with lymph node metastasis risk. Hence, we constructed an immune score-based prognostic nomogram for predicting the presence of lymph node metastasis in PTC.

Immune cells play a key role in tumor progression, and immune cell infiltration is considered a prognostic biomarker for immunotherapy (8). Our findings showed that a higher immune score is significantly related to a higher risk of lymph node metastasis. A high immune score suggests the predominance of immune cell infiltration in the tumor microenvironment. A likely explanation is that the immune microenvironment may be dominated by immunosuppressive cells like macrophages, neutrophils (15), dendritic cells (21) and mast cells (22). Studies have demonstrated that immune cells infiltrating in tumor tissue have both pro-tumor and anti-tumor functions. In papillary thyroid cancer, many infiltrating immune cells like tumor-associated macrophages (TAMs), tumor-associated mast cells (TAMCs), and regulatory T cells (Tregs) would contribute to tumor cell immune escape and be related to poor prognosis. Despite numerical superiority, most immune cells are phenotypically immature and functionally weakened, and effector cells, such as plasma cells (8), are decreased, which may reduce cytotoxic T lymphocyte, B cell and NK cell-mediated cancer cell killing through the release of cytokines (9). Thus, the overall immune response is reduced, leading to increased tumor aggressiveness (23–25). The pro-tumor behavior of infiltrating immune cells may be related to a higher BRAF V600E point mutation rate.

However, we did not successfully verify the clinical characteristics that were previously confirmed to have a significant association with aggressiveness and lymph node metastasis, such as multifocality (26–28). Possible reasons for this include the relatively small sample size, selection bias on the raw data, the different inclusion criteria and study setting compared with previous studies. Thus, the clinical-pathological characteristics, especially those significant during the univariate analysis but not in multivariate analysis, need to be further evaluated by more multicenter studies with larger sample sizes.

The results showed that younger age, larger tumor volumes (T2-4), and nonclassical histological type were relatively more likely to develop lymph node metastasis, which was in line with the findings of previous studies (29–33).

To our knowledge, our study built the first nomogram for predicting lymph node metastasis risk in PTC based on the immune score. This scoring system allows physicians to comprehensively assess the risk of lymph node metastasis in patients and develop treatment plans appropriate for individual patients, including lymph node dissection and I131 therapy.

There were still some limitations that should be mentioned. First, this study made use of TCGA public data, which is a nonrandomized, retrospective study. The TCGA database does not include preoperative cervical ultrasonography, so the analysis was based on pathological staging but not preoperative clinical staging. Additionally, there may have been some information bias and selection bias in the raw data due to the lack of genetic data available for immune score calculation. Second, some of the results in our study differed from those reported in previous studies, which may be related to differences in inclusion criteria and data selection. Third, the univariate and multivariate analyses and nomogram construction relied on logistic regression, which may overestimate the OR value when the incidence of outcome events is high (>10%) (34). Fourth, the nomogram lacks external validation, which limits its clinical application. Hence, more prospective cohorts for external validation are needed to assess the reliability and practicability of our nomogram.

## Conclusion

The high immune score group was significantly correlated with lymph node metastasis. Age <55 years, T-stage, unifocal lesions, and nonclassical PTC were also associated with a higher risk of lymph node metastasis. A nomogram constructed based on immune scores and other clinical-pathological features can help predict lymph node metastasis and has potential clinical application.

## Data availability statement

Publicly available datasets were analyzed in this study. This data can be found here: https://xenabrowser.net/datapages/?cohort=GDC%20TCGA%20Thyroid%20Cancer%20(THCA)&removeHub=https%3A%2F%2Fxena.treehouse.gi.ucsc.edu%3A443.

## Author contributions

KG contributed to the conception and design of the study. KG and YL downloaded and organized the database. YL performed the statistical analysis. YL wrote the first draft of the manuscript. KQ, MF wrote sections of the manuscript. All authors contributed to the article and approved the submitted version.

## Funding

This study was supported by National Natural Science Foundation of China (81972496); Outstanding Academic Leaders Plan, Huangpu District, Shanghai; Adolescents Science & Technology Innovation studio, Shanghai Jiao Tong University School of Medicine.

## Conflict of interest

The authors declare that the research was conducted in the absence of any commercial or financial relationships that could be construed as a potential conflict of interest.

The reviewer SX declared a shared affiliation with the authors to the handling editor at the time of review.

## Publisher’s note

All claims expressed in this article are solely those of the authors and do not necessarily represent those of their affiliated organizations, or those of the publisher, the editors and the reviewers. Any product that may be evaluated in this article, or claim that may be made by its manufacturer, is not guaranteed or endorsed by the publisher.
