# Supplementary figures and images for: A prognostic nomogram for papillary thyroid cancer lymph node metastasis based on immune score
Source: Front Endocrinol (Lausanne). 2022 Dec 1;13:993856. doi: 10.3389/fendo.2022.993856 (PMC9751967; doi:10.3389/fendo.2022.993856)

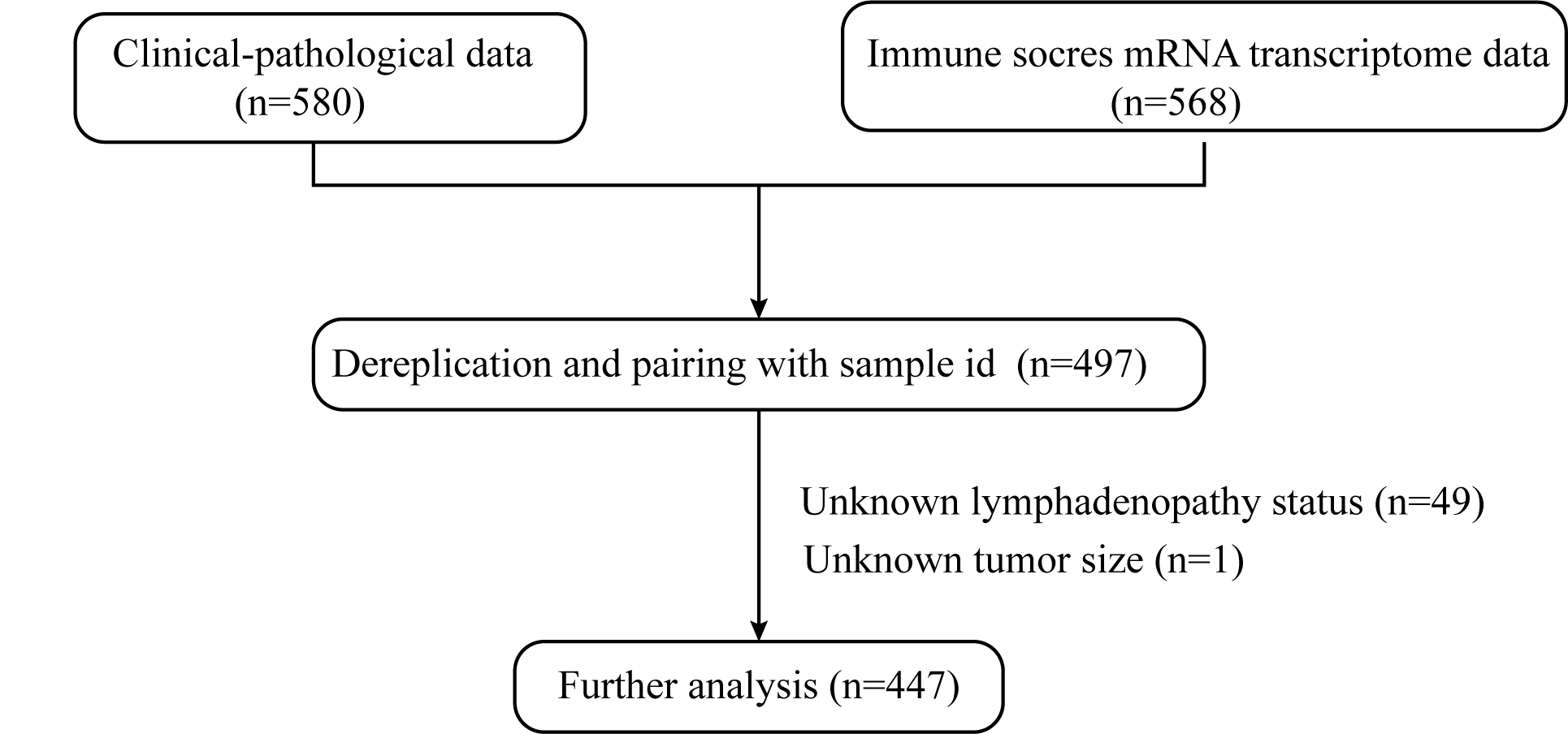

Supplement: Supplementary file 3 [file Image_1.tif]
